# Optimising the classification of feature-based attention in frequency-tagged electroencephalography data

**DOI:** 10.1038/s41597-022-01398-z

**Published:** 2022-06-13

**Authors:** Angela I. Renton, David R. Painter, Jason B. Mattingley

**Affiliations:** 1 The University of Queensland, Queensland Brain Institute, St Lucia, 4072 Australia; 2 The University of Queensland, School of Information Technology and Electrical Engineering, St Lucia, Australia; 3 The University of Queensland, School of Psychology, St Lucia, 4072 Australia; 4 Canadian Institute for Advanced Research (CIFAR), Toronto, Canada

**Keywords:** Attention, Biomedical engineering

## Abstract

Brain-computer interfaces (BCIs) are a rapidly expanding field of study and require accurate and reliable real-time decoding of patterns of neural activity. These protocols often exploit selective attention, a neural mechanism that prioritises the sensory processing of task-relevant stimulus features (feature-based attention) or task-relevant spatial locations (spatial attention). Within the visual modality, attentional modulation of neural responses to different inputs is well indexed by steady-state visual evoked potentials (SSVEPs). These signals are reliably present in single-trial electroencephalography (EEG) data, are largely resilient to common EEG artifacts, and allow separation of neural responses to numerous concurrently presented visual stimuli. To date, efforts to use single-trial SSVEPs to classify visual attention for BCI control have largely focused on spatial attention rather than feature-based attention. Here, we present a dataset that allows for the development and benchmarking of algorithms to classify feature-based attention using single-trial EEG data. The dataset includes EEG and behavioural responses from 30 healthy human participants who performed a feature-based motion discrimination task on frequency tagged visual stimuli.

## Abstract (summary)

**Table:** 

Measurement(s)	Electroencephalography
Technology Type(s)	electroencephalography (EEG)
Factor Type(s)	Cued colour (black, white) • Cued flicker frequency (6.0 Hz, 7.5 Hz) • Distractor (present, absent)
Sample Characteristic - Organism	Homo sapiens
Sample Characteristic - Environment	time stamped measurement datum
Sample Characteristic - Location	Australia

## Background & Summary

Recent advances in machine learning algorithms, computer processing power and neuroimaging hardware have driven significant progress in the field of brain-computer interfaces (BCI). BCI systems apply real-time decoding algorithms to human neuroimaging data, with the goal of extracting reliable patterns of neural activity to operate external devices or provide neurofeedback training^1–4^. Visual selective attention, the brain’s ability to selectively allocate its limited processing resources to a behaviourally relevant subset of visual inputs, is a common target for BCI control^5,6^. By deliberately shifting attentional focus across visual elements in a display, human participants are able to enhance their neural response to some display elements while suppressing their response to others^7,8^. Many BCI applications already take advantage of this phenomenon, allowing users to shift their attentional focus across different spatial locations to communicate and control computer displays^4,9,10^. These shifts in spatial attention produce topographically distinct patterns of neural activity which are readily classifiable^11–13^. Unfortunately, detection of spatial attentional selection typically relies on observers’ continually foveating a fixation spot, either centrally or at the selected spatial location. This requirement prevents participants from visually exploring their environment, or even visually monitoring the controlled object^14,15^. Further, the approach of classifying spatial attention is likely to be inappropriate for patient groups with disordered gaze control, for whom BCIs are often designed^16^. A useful extension of this approach, therefore, would be to have participants prioritise display elements on the basis of selected visual features^5,17^ (e.g. luminance^18^, colour^7^, or motion-direction^19^) or visual objects^20,21^ to control a BCI. Despite the potential benefits of such approaches, feature-based and object-based attention have not been widely used for BCI control, in part because the unique patterns of neural activity associated with attending to specific visual features have proven more difficult to classify using non-invasive neuroimaging techniques^22^. To address this issue, we present a dataset to facilitate the training of algorithms to discern the current focus of feature-based attention in real-time using electroencephalography (EEG).

To track how attention is distributed across multiple concurrent visual stimuli, neuroscientists often use frequency tagging methods^6,8,23–25^. Frequency tagging involves flickering visual stimuli at specific frequencies, thus eliciting time-locked frequency-specific responses from visual system neural populations, termed steady-state visual evoked potentials (SSVEPs)^25,26^. When attention is selectively allocated to a visual stimulus flickering at a specific frequency, the neural response to that stimulus is enhanced, which in turn evokes a larger amplitude SSVEP at the attended flicker frequency^24^. Simultaneously, the amplitudes of neural responses to ignored stimuli remain unchanged, or may even be suppressed^7,27^. SSVEPs can allow continuous tracking and quantification of the deployment of attention across multiple concurrent stimuli. Although SSVEPs have been widely adopted to control BCIs via spatial attention^10,12,28^, there has been much less work on BCI control using feature-based attention^22,29^. To date, there is no open dataset specifically designed to facilitate the development and training of machine learning algorithms to classify the target of feature-based attention using single-trial frequency-tagged EEG data. The development of such algorithms would have wide ranging applications, from basic visual neuroscience^1,30,31^, through to the development of neurofeedback training protocols for clinical and research applications^29,32–34^ and BCIs that allow participants to control display elements or robotic devices^22,35–37^ through attentional control.

To this end, we collected EEG and behavioural data from healthy adult participants as they monitored random-dot kinematograms (RDKs) made up of dynamically moving black and white dots. Participants were cued to identify target bursts of coherent motion in dots of one colour (the attended stimulus) while ignoring dots in the other colour (the distractor stimulus). Throughout each trial most of the dots in each of the coloured sets moved randomly, making the periods of coherent motion relatively difficult to discern. Such motion discrimination tasks have been widely employed to study perceptual decision-making in both humans and other animal species^38–40^. We had participants engage their feature-based attention to monitor dots of one colour, while filtering out the irrelevant information from the remaining dots. We also introduced a condition in which dots of only one colour were presented, designed to simulate complete, deliberate attentional suppression of the distractor colour in the training data. We posited that training classifiers on this condition might mitigate overfitting effects by overemphasising amplitude differences in SSVEPs as the differentiating feature between classes. An important design consideration for the task used to generate this dataset was that it should always be possible to distinguish between “top-down” and “bottom-up” effects on SSVEPs^41^. Feature-based attention controlled BCIs depend upon the classification of top-down effects, generated when participants exert attentional control to select one feature over another. However, SSVEPs are also affected by stimulus driven factors such as the size, contrast, colour, speed, and retinotopic position of a flickering object^26^. As such, every effort was made to keep such stimulus factors constant across the flickering dots of each colour, such that SSVEP amplitudes could only be affected by top-down feature-based selection.

## Methods

### Participants

Thirty-two healthy adult participants (16 males, age *M* = 23.43 years, *SD* = 5.44 years) volunteered for the experiment after providing written informed consent, and each was paid $20 for attendance. The study was approved by The University of Queensland Human Research Ethics Committee and was performed in accordance with the relevant guidelines and regulations. Participants provided informed written consent to have their de-identified data made open access to the scientific community. Data from two participants were excluded from the dataset due to technical difficulties that corrupted the EEG recordings.

### Task overview

**Fig. 1:**
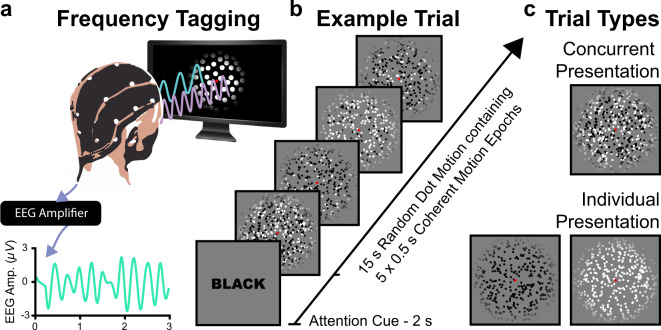
Schematic of experimental task and structure of a typical trial. (**a**) Participants performed a dot motion discrimination task on a set of flickering dots while we recorded continuous EEG data. (**b**) Each 17 s trial consisted of a 2 s cue (‘BLACK’, ‘WHITE’) to indicate which colour participants should focus on, followed by a 15 s period which contained 5 coherent motion targets. (**c**) Different coloured dot patches were presented either concurrently (distractor present) or individually (distractor absent). When the distractor was present, black and white dots, flickering at 6.0 & 7.5 Hz (colour and frequency were counterbalanced) appeared intermingled at the same spatial location, and participants attended only to motion in dots of one colour. When the distractor was absent, dots of only one colour (black or white), flickering at one frequency, were presented.

The task was designed to generate a dataset on which to train machine learning classifiers to discriminate between attended and unattended features. Participants were tasked with monitoring a field of randomly moving, flickering dots in order to identify short bursts of coherent motion in a cued colour (black or white, Fig. 1a). To establish a ground truth for the target of feature-selective attention, coherent motion events were only presented in the cued colour. Supervised machine learning classification is fundamentally contingent upon accurate data labelling. While it may be impossible to know the true state of feature-selective attention corresponding with every sample of EEG data, this design choice meant that there would be no epochs during which attention had been captured to the non-target colour by a distractor motion event. The fields of black and white dots were entirely co-mingled in space, such that any shifts in gaze would equally affect SSVEPs at both frequencies. Note that we varied the luminance of these dots such that they appeared as either black or white against the intermediate grey background. Thus, the manipulation was strictly one of luminance (decrement or increment), but for simplicity of exposition we refer to these here as different colour categories (black or white). This design choice meant that the features could be discriminated by colour-blind individuals. It is also worth noting that the fields of black and white dots might be perceptually grouped into two visual objects, at least by some observers. As such, these data may be applicable to the machine learning classification of both feature-based and object-based attention^20,42^. A cue presented before each 15 s trial indicated whether participants should monitor the black or the white dots on each trial (Fig. 1b). Bursts of coherent motion occurred when a subset of the dots moved in the same direction (up, down, left, or right) for 500 ms. During half the trials, black and white dots were presented concurrently (distractor present), and participants were cued to monitor for bursts of coherent motion in only one of the two colours. During the remaining trials, only the cued coloured dots were presented (distractor absent), simulating the complete, deliberate attentional suppression of the uncued colour (Fig. 1c). Dots in different colours flickered at different frequencies (6.0 Hz, 7.5 Hz; colour and frequency were counterbalanced), thus evoking SSVEPs. When only one colour was present, SSVEPs were only evoked at a single frequency counterbalanced across trials.

#### Trial design

Each trial lasted 17 s, and was composed of a 2 s cue followed by 15 s of dot motion (Fig. 1b). This relatively long trial period was chosen to allow for sliding window decoding of the current target of feature selective attention over prolonged periods of constant stimulation, a desirable feature for BCI control. Participants should be able to switch their feature-selective attention between two stable targets indefinitely, and previous work suggests that the effects of feature selective attention on SSVEP amplitudes remain discriminable for at least the duration of 15 s trials^6^. Each 15 s period of dot motion contained five 500 ms bursts of coherent motion (targets). Coherent motion onset times were randomly selected for each trial, with the constraint that they occurred more than 1 s after display onset, more than 1.5 s after the offset of the previous motion epoch, and more than 1.5 s before the end of the trial. Coherence for all motion targets was set to 50%. Thus, for each coherent motion epoch, 50% of the dots moved either up, down, left, or right across the display, and the remaining 50% of dots moved in random directions. The direction of coherent motion was counterbalanced such that each direction occurred an equal number of times for each distractor condition (present, absent) and each flicker frequency (black - 6.0 Hz, white - 7.5 Hz; black - 7.5 Hz, white - 6.0 Hz). Participants were asked to respond to motion targets as quickly and accurately as possible by pressing the arrow on the keyboard corresponding to the perceived motion direction ([↑], [↓], [←], [→]). To avoid inadvertently drawing participants’ attention to the uncued feature, coherent motion targets only occured in the cued colour.

#### Block design and counterbalancing

The experiment consisted of 160 trials, organised into 8 blocks of 20 trials. Distractor (present, absent), cue colour (black, white) and flicker frequency (6.0 Hz, 7.5 Hz) were all fully counterbalanced. Thus, the distractor was present on 50% of trials (both black and white dots presented), and absent on the remaining 50% of trials (either black dots or white dots presented alone). Within each of these trial types (distractor present, distractor absent), black and white dots were cued equally often. Dots of the cued colour flickered at 6.0 Hz and 7.5 Hz on an equal number of trials. On distractor present trials, the uncued colour flickered at the remaining frequency (i.e. cued colour: 6.0 Hz, uncued colour: 7.5 Hz; cued colour: 7.5 Hz, uncued colour: 6.0 Hz).

#### Frequency tagging design

Motion coherence for the motion targets was set to 50%, while the remaining 50% of dots moved in random directions. Critically, flicker was limited to those 50% of dots which were never used to form coherent motion. As such, motion events could not alter the frequency-tags, preventing any bottom-up signal from aiding classification of the cued colour. The randomly moving dots oscillated in a square wave flicker with a 50% duty cycle to induce frequency tags. Black flickering dots oscillated between RGB: 0 and RGB: 128, and white flickering dots oscillated between RGB: 128 and RGB: 255. The flicker frequencies (6.0 Hz, 7.5 Hz) were chosen such that the SSVEPs and any harmonics would be outside the alpha range. Further, these flicker frequencies were likely to evoke high amplitude SSVEPs^43^ and had been demonstrated to be sensitive to feature-selective attention^6,24,25^.

#### Stimulus design

Each moving dot subtended 0.27° × 0.27° of visual angle, and moved at a rate of 1.49° of visual angle/sec across the display, within a circular area with a radius of 5.20° of visual angle. Dots which moved beyond the boundary of this circular area immediately reappeared 180° from the point of disappearance, maintaining the same angular trajectory. There were 400 moving dots of each colour (black, white), and thus 800 moving dots in total on distractor present trials, and 400 moving dots in total on distractor absent trials. The starting position of each dot was randomly selected on each trial, with the constraint that it was within the circular display area. The drawing order of all 800 dots was also randomised across trials, such that any dot was as likely to appear above the others as below. On each trial, the movement direction of each of the 800 dots was sampled from a uniform distribution spanning 0–360°. For each colour, 50% of these moving dots flickered to induce a frequency tag and maintained their randomly sampled trajectory throughout the trial. The remaining 50% of dots did not flicker, and periodically moved coherently in one of four directions (0°, 90°, 180°, 270°). A red (RGB: 255, 0, 0) fixation cross which subtended 0.12° × 0.12° of visual angle was presented at the centre of the display, on top of all the moving dots. At the beginning of each trial, a cue in 60 pt. Arial font was presented in the centre of the screen, indicating whether participants should attend to the black (“BLACK”) or white (“WHITE”) dots. The colour of the cue text matched the cue condition. At the beginning of each block of trials, a screen announced which block would follow (e.g., “BLOCK 1”) in black 40 pt. Arial font. After the first block, this screen also announced the participant’s average behavioural accuracy and reaction time for the previous block of trials. After an enforced 10 s break, the words “Press [ENTER]” appeared to indicate that participants could proceed to the next block by a button press when ready. The experiment was presented on a uniform grey (RGB: 128, 128, 128) background.

#### Display computer specifications

The display was presented at a viewing distance of 57 cm on a 24-inch ASUS VG248QE monitor with a refresh rate of 120 Hz and resolution of 1920 × 1080. Stimuli were presented using PsychToolbox-3 (Kleiner *et al*., 2007) running in MATLAB R2017a (64-bit) under Windows 10 (64-bit). The experiment was run on a Dell Precision Tower 5810 desktop computer containing an Intel Xeon E7-4809 v2 CPU and NVIDIA QUADRO M4000 GPU.

### EEG Recording and processing

#### EEG recording

EEG data were sampled at 1200 Hz using a g.USBamp amplifier (g.tec Medical Engineering, GmbH, Austria) from 5 active Ag/AgCl scalp electrodes arranged according to the international standard 10–20 system for electrode placement in a nylon head cap (Oostenveld and Praamstra, 2001). The electrode positions, which were clustered symmetrically over occipital brain regions at the back of the head, were as follows: Iz, O1, O2, Oz, and POz. This minimalist electrode configuration was chosen for efficiency in applications involving large scale data collection. This occipitoparietal electrode configuration covers the area at which SSVEPs are most often maximal^6,10,23^. The ground electrode was positioned at FCz, and an active Ag/AgCl earclip electrode was used as the reference. EEG data were filtered in real time with a notch filter at 48–52 Hz and a 1–100 Hz bandpass filter.

## Data Records

### Distribution for use

The data files for the feature-based attention classification dataset can be accessed through the Open Science Framework as well as via the University of Queensland eSpace data deposition service, and are stored in the BIDS-EEG format^44^ (version 1.0.2) (10.17605/OSF.IO/C689U)^45^. The data repository contains data from 30 participants (7.8 GB), as summarised in Table 1.

**Table 1:** Participant metadata.

ID	Age	Gender	Max Classification Acc (%)	Best Classifier
sub-01	21	Male	61.62	LDA - Present
sub-02	42	Female	63.87	SVM - Present
sub-03	34	Male	82.63	LDA - Present
sub-04	34	Male	83.84	LDA - Present
sub-05	20	Male	57.65	LDA - Present
sub-06	21	Female	—	—
sub-07	23	Female	66.74	LDA - Present
sub-08	24	Male	57.75	LDA - Present
sub-09	21	Male	54.07	SVM - Absent
sub-10	21	Female	70.86	LDA - Present
sub-11	27	Male	90.89	LDA - Present
sub-12	18	Female	68.91	LDA - Present
sub-13	22	Female	57.18	KNN - Absent
sub-14	22	Female	69.07	LDA - Present
sub-15	19	Other	59.13	LDA - Present
sub-16	22	Female	59.25	LDA - Absent
sub-17	—	Female	81.56	LDA - Present
sub-18	18	Male	59.03	SVM - Present
sub-19	20	Male	—	—
sub-20	20	Male	65.98	LDA - Present
sub-21	21	Male	60.85	LDA - Present
sub-22	19	Female	73.84	LDA - Present
sub-23	22	Female	72.26	LDA - Present
sub-24	23	Male	59.16	LDA - Present
sub-25	23	Male	62.27	LDA - Present
sub-26	23	Male	72.26	LDA - Present
sub-27	18	Female	65.05	LDA - Present
sub-28	23	Female	75.41	LDA - Present
sub-29	24	Male	69.21	LDA - Present
sub-30	22	Female	57.02	KNN - Present
sub-31	22	Male	64.27	LDA - Present
sub-32	34	Female	66.37	LDA - Present

Note: the “Best Classifier” column indicates the classifier that was used to achieve the maximal classification accuracy for each participant, and whether data from the distractor “Present” or “Absent” condition were used to achieve this accuracy.

### Overall folder structure

**Fig. 2:**
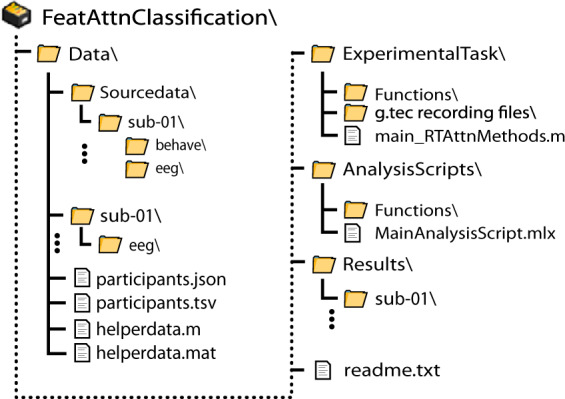
Folder structure of the online data repository. This repository contains four sub-folders. The “Data” folder contains all data and metadata and is organised according to the BIDS-EEG format (version 1.0.2). The “ExperimentalTask” folder contains all code used to run the experimental task and record the associated data. The “AnalysisScripts” folder contains all the scripts used for technical validation of this dataset. Finally, the “Results” folder contains the data files output during technical validation, allowing group aggregate results to be calculated.

The data repository for the feature-based attention classification dataset contains four top-level folders (Fig. 2). Top-level folders include “*FeatAttnClassification\ExperimentalTask\*”, which contains the MATLAB code used to run the experimental task, “*FeatAttnClassification\Data\*”, which contains all EEG and behavioural data, “*FeatAttnClassification\AnalysisScripts\*”, which contains the code used for technical validation (see below), and “*FeatAttnClassification\Results*\”, which contains the files output by the analysis scripts. The data folder follows the BIDS specification for folder hierarchy^44^. Critical information regarding the experimental task parameters, display settings, EEG recording settings and triggers is contained in the file “*FeatAttnClassification\Data\helperdata.mat*”. These values are also described above in the Methods section.

### Experimental task organization

The main script used to run the experimental task is stored in “*FeatAttnClassification\ExperimentalTask\main_RTAttnMethods.m*”. This script relies on a number of functions, which are stored in “*FeatAttnClassification\ExperimentalTask\Functions\*”. These functions include “*SetupDotCoords.m*”, which generates the screen coordinates for each dot in the experiment, “*flipper.m*”, which controls the screen flip and triggering, and “*BlockScreen.m*”, which calculates a running average of participants’ performance and presents this information during the break between each block. During the experiment, EEG data were recorded through the g.tec MATLAB API each time this experimental task script was run for a new participant. The MATLAB scripts for interfacing with this API to read and write the data are stored under “*FeatAttnClassification\ExperimentalTask\g.tec recording files\”*.

### EEG Data organization

#### EEG Data files

The EEG data are organised according to the BIDS architecture within the “*FeatAttnClassification\Data\” folder*. Unprocessed raw data are stored for each participant in a.mat MATLAB data format within “FeatAttnClassification\Data\sourcedata\sub-*\eeg\” (Fig. 2). These.mat files were converted to the BIDS compatible Brain Vision format (.vhdr,.eeg,.vmrk) and stored for each participant within “FeatAttnClassification\Data\sub-*\eeg\” (Fig. 2). Raw data were recorded with an online digital high pass filter at 1 Hz and low pass filter at 100 Hz. Note that this online high-pass filtering procedure may result in small shifts in variance forward in time in the signal, and thus a degree of caution should be exercised in interpreting the exact timing of decoding using these data^46^. No further pre-processing has been applied to the data. The EEG files provided for each participant are outlined in Table 2.

**Table 2:** Description of the EEG data files provided for each participant.

Filename	Description
sub-*_task-FeatAttnDec_eeg.vhdr	*Header file*: This is a text file containing recording parameters and other metadata. This file is typically required for loading Brain Vision formatted data.
sub-*_task-FeatAttnDec_eeg.eeg	*Raw EEG data file*: This is a binary file containing the EEG and trigger event data.
sub-*_task-FeatAttnDec_eeg.vmrk	*Marker file*: This is a text file which is sometimes required for loading Brain Vision formatted EEG data. These files usually contain information describing the trigger events that have been collected during the EEG data recording but are empty in this case.
sub-*_task-FeatAttnDec_eeg.json	*Metadata file:* Specifies details of experimental task and EEG recording (e.g. sampling frequency, power line frequency, filters)
sub-*_task-FeatAttnDec_events.tsv	*Events file:* Lists the timestamps and trigger codes for all events in the recording session.
sub-*_task-FeatAttnDec_channels.tsv	*Channels file:* Provides further information about the raw EEG data as well as information not present in the raw EEG data file such as filter settings and channel status (good/bad).

Note: The * character is used as a wildcard, as filenames include each participant’s unique identifier.

#### EEG Triggers

Triggers marking the onset of pertinent trial events in the EEG data are stored for each participant both in their raw form, in the “TRIG” channel saved to the “sub-*_task-FeatAttnDec_eeg.eeg” file, and in their processed form, in the “sub-*_task-FeatAttnDec_events.tsv” file. Triggers were sent to the amplifier via the parallel port, as integer values between 0 and 255 (8-bit range). These values were converted to voltages; thus, in their raw form, events are marked by increases in voltage amplitude of the “TRIG” channel from the 0 *uV* baseline (range: 0-255 *uV*). The amplitude of this increase marks the value of the trigger, while the sample at which the increase occurs marks the event onset. The processed events file for each participant lists each of these events with the timestamp (in seconds) at which they occurred and the amplitude of the trigger.

Triggers were sent for each trial to mark the onsets of the cue, the trial, each motion event, and feedback presentation. For each of these events, the triggers were set to different values to represent the nature of the trial. The values of these triggers are stored in the file “Data\helperdata.mat”, within the data structure “HELPER.TRIGGERS”. For both the cue onset (HELPER.TRIGGERS.cueonset) and trial onset (HELPER.TRIGGERS.trialonset), these values were stored in a three-dimensional matrix (flicker frequency × colour × presentation type) in which the first dimension represented the flicker frequency of the cued dots on that trial (6.0 Hz or 7.5 Hz), the second dimension represented the colour of the cued dots on that trial (black or white), and the third dimension represented the distractor condition for that trial (present or absent). Thus, for example, to find the timestamps of all trial onsets for trials in which the cued dots flickered at *6.0* *Hz*, the cued colour was *black*, and the distractor was *absent*, one would search for the trigger values stored in HELPER.TRIGGERS.cueonset(1,1,2). That is, the first value along the first dimension (flicker frequency; *6.0* *Hz*, 7.5 Hz), the first value along the second dimension (colour; *black*, white), and the second value along the third dimension (distractor; *present, absent*). The organisation of the triggers marking motion epoch onsets (HELPER.TRIGGERS.motiononset) is similar, with the exception that a fourth dimension is added to the matrix, which marks the motion direction presented during that epoch (0°, 90°, 180°, 270°). The feedback trigger was always 222.

### Behavioural data organisation

Behavioural data are stored for each participant under “FeatAttnClassification\Data\sourcedata\sub-*\behave\sub-*_task-FeatAttnDec_behav.mat”. These files contain all the variables created when the experimental task was run for each participant. The four variables critical for any behavioural analysis are listed in Table 3.

**Table 3:** Description of the data containers used to store the key behavioural information for each participant.

Variable	Description
**TRIAL_TABLE**	TRIAL_TABLE is a MATLAB Table data type describing the attributes of each trial. Each row represents a trial in the experiment. The columns are as follows: • BLOCK: The experimental block number (1–8). • TRIAL: The trial number (1–160). • ATTENTIONCOND: The presentation type of the dots (1 = distractor present, 2 = distractor absent). • col_Attd_UnAttd: This is a 2D array. The first column represents the cued colour, and the second column represents the uncued colour (1 = black, 2 = white). • Freq_colA_colB: This is a 2D array. The first column represents the flicker frequency of the black dots, and the second column represents the flicker frequency of the white dots (1 = 6.0 Hz, 2 = 7.5 Hz). • movedir_attd_UnAttd: This is a 2D cell array. The first cell in each row represents movement directions for the cued dots. The second cell contains movement directions that were generated for the uncued dots but were not presented (a legacy parameter). Each cell contains 5 values sequentially representing the motion directions of the 5 motion targets for that trial. • movedir_colA_colB: This is a 2D cell array. The first cell in each row represents movement directions generated for the black dots, the second cell represents movement directions generated for the white dots. Note that motion targets were only presented in the cued colour on each trial. Each cell contains 5 values sequentially representing the motion directions of the 5 motion targets for that trial. • moveframe_AttdUnAttd: This is a 2D cell array. The first cell in each row represents movement onsets for the cued dots. The second cell in each row contains movement onsets that were generated for the uncued dots but were not presented. Each cell contains 5 values, sequentially representing the frame indices for the onsets of each of the 5 motion targets for that trial. • moveframe_colA_colB: This contains two sub-columns. The first represents movement onsets generated for the black dots, the second represents movement onsets generated for the white dots. Note that motion targets were only presented in the cued colour on each trial. Each cell contains 5 values, sequentially representing the frame indices for the onsets of each of the 5 motion targets for that trial.
**RESPONSE**	RESPONSE is a 2D matrix (no. trials × no. frames) in which the columns represent individual trials and the rows represent the frames in those trials (screen refresh rate = 120 Hz, trial duration = 15 s). The values of this matrix represent the key press responses to the perceived motion direction during each frame of each trial (0 = no key pressed, 1 = 0°, 2 = 90°, 3 = 180°, 4 = 270°).
**ACC**	ACC (%) is a 2D matrix (no. trials × no. targets/trial) in which the columns represent individual trials and the rows represent motion targets within that trial. The values of this matrix represent the accuracy of the participants’ responses to these motion targets (0 = miss, 1 = correct response, 2 = incorrect response).
**RT**	RT (s) is a 2D matrix, (no. trials × no. targets/trial) in which the columns represent individual trials and the rows represent motion targets within that trial. The values of this matrix represent the reaction time for motion targets on which a correct response was made. Reaction times were taken from the onset of the coherent motion epoch to the onset of the participant’s key press.

### Analysis file organisation

The scripts used to run the analyses reported in the technical validation section below are stored in “*FeatAttnClassification\AnalysisScripts\*”. The subfolder “*FeatAttnClassification\AnalysisScripts\Functions*” contains functions which are called across several of the analysis scripts. The function of each analysis script is described in Table 4.

**Table 4:** List of analysis scripts used for technical validation.

Filename	Description
Main_Decodingt.mlx Main_Decoding.pdf Main_Decoding.html	Matlab live script containing the main implementation of the data loading, pre-processing, visualisations, and application of the machine learning classifiers. The Matlab live script has also been output to.pdf and.html formats for ease of viewing.
Main_Decoding.m	The standard.m MATLAB script format of MainAnalysisScript.mlx
Aggregate_Decoding.m	This script collates the results produced by all other scripts to generate the group average results and comparisons across training types.
Aggregate_Behave.m	This script performs behavioural analyses for each participant.
Aggregate_SSVEPs.m	This script performs the analyses on the SSVEP amplitudes.

### Results file organisation

The data files generated by the analysis scripts run on each individual participant are stored under “FeatAttnClassification\Results\\sub-*\”. The files generated for each participant are described in Table 5.

**Table 5:** List of data files generated as the results of the analysis scripts for each individual participant.

Filename	Description
S*BehaveResults.mat	Behavioural results for the motion discrimination task, including mean accuracy (%) and reaction time (s) by distractor and colour cue, as well as performance for each individual motion epoch.
S*ACCURACY_*_Train*TestMultifreq.mat	Files containing the accuracy (%) for each classifier (z-score, logistic regression, linear discriminant analysis, support vector machine, multi-layer perceptron, k-nearest neighbours), when trained on distractor absent and distractor present data.

## Technical Validation

We designed the current study to create a dataset that would allow the scientific community to design and benchmark different approaches to real-time feature-based attention classification. To validate the suitability of the dataset for this purpose, we compared the efficacy of six different algorithms for classifying the target of feature-based attention, using different combinations of training features. We compared training on distractor absent trials, in which only a single-coloured dot field was visible, with training on distractor present trials, in which both coloured dot fields were visible concurrently. This allowed us to assess whether training on a simulated template of complete attentional suppression of the unattended item might improve classification accuracy. Finally, we modelled how increasing the size of the sliding window impacts classification accuracy across these approaches. In order to share the technical validation of the dataset with maximal transparency, we have shared the scripts used to train and test our classifiers as MATLAB Live notebooks (an interactive format which enables code blocks to be intermingled with text, images, equations, and script outputs from print statements and plots). Note that the results described here are merely intended to demonstrate the suitability of this dataset for benchmarking feature-based attention classification algorithms. The findings presented do not represent the full range of approaches that could be applied to classify which visual features are attended in real-time, and there are as yet many unexplored avenues toward feature-based attention controlled BCIs. Our hope is that this dataset will facilitate the discovery and development of these avenues.

### Description of analyses

#### Data sorting

**Fig. 3:**
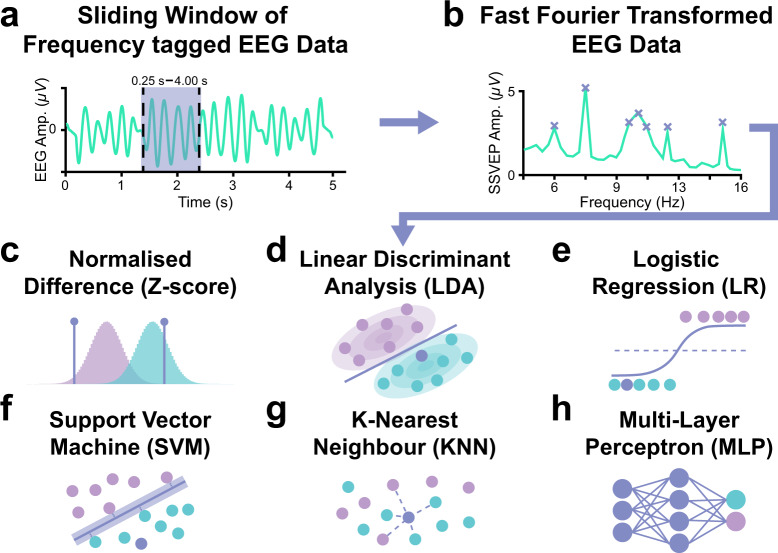
Machine learning pipeline for classification of the attended feature in the motion-discrimination task. (**a**) Sliding window EEG data were extracted for each trial using five different sliding window sizes (0.25 s, 0.50 s, 1.00 s, 2.00 s, 4.00 s) sampled at 0.25 s. (**b**) These epochs were zero-padded out to a minimum of 2 s to achieve 0.5 Hz spectral resolution across all window sizes and were submitted to FFTs. For each epoch, the FFT amplitude for each channel of EEG was extracted at the flicker frequencies (6.0 & 7.5 Hz), the frequencies in the alpha range (8–12 Hz), and the second harmonic of the flicker frequencies (i.e., 12.0 & 15.0 Hz). The FFT amplitudes across the relevant frequencies were used to classify the attended colour for each epoch using classification algorithms based on (**c**) a baseline normalised difference approach derived from first principles, (**d**) linear discriminant analysis (LDA), (**e**) logistic regression (LR), (**f**) support vector machine (SVM), (**g**) *k*-nearest neighbours (KNN), and (**h**) multi-layer perceptron.

Before we began training and testing our classifiers, the raw data were processed and sorted. Each 15 s epoch of EEG data in a trial was extracted and labelled according to the distractor condition (present, absent) and colour-flicker frequency condition (black: 6.0 Hz and white: 7.5 Hz, black: 7.5 Hz and white: 6.0 Hz). For each combination of these conditions, we aimed to classify the cued colour (black, white). Note that this split was performed because we expected that SSVEP amplitudes would vary as a function of both the flicker frequency and colour of attended dots. While differences on the basis of colour may be more subtle for more equiluminant colour-pairs, differences in response should always be expected due to individual differences in chromatic channels^47,48^. To compare classification accuracy across different sliding-window sizes, we extracted epochs of data from these trials using five different sliding window sizes (0.25 s, 0.50 s, 1.00 s, 2.00 s, 4.00 s) sampled at 0.25 s intervals (Fig. 3a). Epochs in which the absolute value of the EEG amplitude in any channel was greater than 150 *µ*V were excluded from training and testing, as these were likely to be motion artifacts^49^. Further, we defined a 1 s period spanning from the onset of each period of coherent motion to the point at which the event related activity locked to this onset had returned to baseline. Epochs for which more than 33% of the data fell within this period were also excluded from training and testing. This was done so that the classifiers could not rely on any bottom-up differences driven by the onsets of coherent motion, but rather had to detect differences in the SSVEPs driven by top-down engagement of feature-based attention^41^. Finally, we ensured that data were still equally distributed across conditions (attend black, attend white) after these epochs were removed by randomly removing additional epochs from the larger class until epoch numbers were balanced. For the remaining data, epochs less than 2 s in length were zero-padded out to 2 s to achieve a minimum of 0.5 Hz spectral resolution for all sliding window sizes. These epochs of EEG data were each submitted to a fast Fourier transform (FFT, Fig. 3b). The amplitude of the FFT at the tagged frequencies, their second harmonics, and each available frequency in the alpha range (6.0 Hz, 7.5 Hz, 12.0 Hz, 15.0 Hz, 8.0–12.0 Hz) were then extracted for every EEG channel and stored in a single feature vector.

#### Training conditions

We trained the machine learning classifiers under a number of different conditions to assess how these affected classification accuracy. Primarily, we compared how training on distractor present trials compared with training on distractor absent trials. We also assessed how classification accuracies changed with increasing epoch durations (0.25 s, 0.50 s, 2.00 s, 4.00 s). To assess how different combinations of features affected classification accuracy, we trained the classifiers initially using just the first harmonic frequencies (‘simple’). Next, we asked whether the second harmonic might contribute unique variance to aid classification by including both the first and second harmonic frequencies (‘simple + harmonic’)^25^. Third, given that endogenous alpha oscillations have been linked to sustained attentional processes, which may interact with feature-selective attention, we trained classifiers using both the first harmonic and alpha frequencies (‘simple + alpha’)^50^. Finally, we trained the classifiers using spectral power at both the first and second harmonic frequencies as well as alpha activity (‘simple + alpha + harmonic’).

#### Testing conditions

The experiment was designed to determine how best to classify which of two visual features a participant is attending to, using short latency, single-trial EEG data. Our primary test condition was therefore classification on the distractor present trials. When training was performed on the distractor absent trials, classification accuracy for each participant was determined using a single iteration of training on distractor absent trials and testing on distractor present trials (as there was no overlap between these sets of trials). When training was performed on the distractor present trials, classification accuracy for each participant was determined using 10-fold cross validation across trials (i.e. separation was performed on the basis of trial number, such that train- and test-data always represented independent trials)^51^.

### Machine learning approaches

Several approaches were used to discriminate which feature participants were attending to using short latency single-trial EEG data from a low density electrode array positioned over the posterior scalp. These included a *z*-score difference approach, linear discriminant analysis, logistic regression, support vector machine, multi-layer perceptron, and k-nearest neighbours.

#### Z-Score difference

We first set out to determine baseline classification accuracy using an approach derived from first principles. It has been well established that SSVEP amplitude at any given frequency typically increases when the driving stimulus is attended, and decreases when the driving stimulus is actively suppressed by attention^8,24^. Nevertheless, the baseline amplitude of the SSVEP and the size of any attention-induced changes in SSVEP amplitude differ according to a number of factors such as individual participant fatigue or habituation, flicker frequency, stimulus properties, and gaze position. This means that the raw difference in single-trial SSVEP amplitudes for two different stimuli/frequencies is not, by itself, a perfect indication of whether one has been allocated more attentional resources than the other. To account for this, we normalised SSVEPs for each frequency (6.0 Hz, 7.5 Hz). This normalisation was performed independently for epochs in each combination of distractor condition (present, absent) and colour-flicker frequency condition (black: 6.0 Hz and white: 7.5 Hz, black: 7.5 Hz and white: 6.0 Hz). That is to say, we generated separate populations of SSVEP amplitudes for each participant representing the response at each flicker frequency given each unique display configuration. For each epoch, we *z*-scored the SSVEPs using the relevant populations (e.g., black: 6.0 Hz, white, 7.5 Hz, distractor present). The driving stimulus with the largest *z*-score for each epoch was classified as attended (Fig. 3c).

#### Linear discriminant analysis (LDA)

While the *z*-scoring approach was derived entirely from first-principles knowledge of SSVEPs, the simple implementation presented above does not take advantage of the high dimensional data available (SSVEP amplitudes at multiple frequencies, available at multiple different electrodes). Linear discriminant analysis (LDA) involves determining the linear boundary in multi-dimensional space which best separates the distributions of features for two classes^52^. Having determined this boundary, we can ask whether each new sample falls closer to the mean of class 1 (attend black) or class 2 (attend white), along the linear boundary separating these two means in multidimensional space. This relatively simple approach builds naturally on the linear *z*-scoring difference rule, while taking advantage of the multi-dimensional data. Notably, an LDA approach has been applied as part of a successful SSVEP-based feature-based attention controlled BCI^29^, and is also a popular choice for BCI control in other contexts^53–55^, suggesting that this approach is likely to perform well for this use-case. We applied LDA to the feature vector containing the SSVEP amplitudes at each EEG channel and relevant frequency using the *fitcdiscr* function from the MATLAB statistics and machine learning toolbox (Fig. 3d).

#### Logistic regression (LR)

LDA-based classification is an attractive option for BCI control as the algorithm has a closed-form analytical solution, and is therefore able to achieve robust performance given relatively low training times. However, this method relies on normally distributed data with equal covariance across classes, considering the entire distribution of features. As such, these classifiers are susceptible to bias when the distribution of training data contains outliers or skew^56^. This may be particularly relevant to the case of classifying single-trial SSVEPs, which typically form a highly skewed distribution. Logistic regression (LR) algorithms represent an alternative linear classification option which circumvent this issue. Much like LDA, LR approaches to classification involve identifying the linear boundary between features associated with each class. Indeed, the two algorithms have the same functional form, but differ in their approach to coefficient estimation; where LDA relies on the assumptions of normality and homoscedasticity, the coefficients in the LR model are estimated using maximum likelihood estimation^57^. As such, LR models make no assumptions about the underlying distributions of predictor variables. However, it is worth noting that the features generated for this technical validation likely violate the logistic regression assumption of lack of multicollinearity amongst predictors, as SSVEPs were calculated for neighbouring electrodes. This limitation could be overcome by the application of principal components analysis (PCA), a common pre-processing step for machine learning applications^58,59^. This approach was not applied here to conserve consistency across methods, but is worthy of future investigation. We applied an LR approach to classification using the *fitclinear* function from the MATLAB statistics and machine learning toolbox (Fig. 3e). Optimisation was performed using stochastic gradient descent (SGD). To avoid overfitting, we applied L2 regularisation with *Lamda* *=* *1/n*.

#### Support vector machine (SVM)

Support vector machine (SVM) algorithms represent another alternative linear classification option which has been shown to perform well with non-parametric EEG features^60,61^. As such, this algorithm represents a popular method for BCI control^62–64^. SVM algorithms optimise the linear margin between features of each class to identify the margin which results in the least possible overlap between classes. Rather than relying on the entire distribution of features, optimisation is performed using only those data points which lie closest to the class boundary (the so-called support vectors). An advantage of this method is that it can be extended to classify data with non-linear boundaries via the application of a kernel function that transforms feature-data into a higher dimensional space in which classes are linearly separable^65^. We applied this transformation using the popular radial basis function (RBF) kernel^66–68^. SVMs with RBF kernels were trained on the feature vectors using the *fitcsvm* function from the MATLAB statistics and machine learning toolbox (Fig. 3f). The RBF kernel scale was automatically selected for each fold using a heuristic subsampling procedure built into the MATLAB *fitcsvm* function.

#### Multi-layer perceptron (MLP)

The difference in EEG signals between attending to dots flickering at one frequency as opposed to the other is likely to involve complex changes in the both the amplitude and topographical distribution of neural signals, and it is thus possible that the difference between classes will not be well characterised by linear discrimination. Multi-layer perceptrons (MLP) are able to map out complex state spaces by weighting each individual input feature across hidden layers to best discriminate between the output classes^69^. We therefore generated fully connected MLPs, with two hidden layers containing 10 nodes in each hidden layer, using the MATLAB Statistics and Machine Learning Toolbox. The network was initialised using the *patternnet* function with the training function set to *traingdx*, which implements gradient descent with momentum and an adaptive learning rate^70^. When training machine learning classifiers, we are typically trying to find the lowest possible point on a “loss function”, which describes the relationship between each hyperparameter and the error we would expect from the model (this process is called optimisation). For neural networks, the loss function typically describes a vast multidimensional landscape with no algorithmic solution to the optimisation problem. SGD algorithms perform optimisation by calculating the gradient (or slope) of the loss function at each training step for the current hyperparameters. In a process known as backpropagation, these hyperparameters can then be adjusted in the direction that is projected to lead to a smaller loss on the next training run. The size of the change made to the hyperparameters in each training run is known as the learning rate. This process can be pictured like a ball rolling down through a mountain range toward the lowest possible loss value. While this approach is effective and widely used^71^, our hypothetical rolling ball is often caught by craters in the mountain-side (i.e. local minima) making no further progress down the loss function. This can be overcome by adding momentum to our rolling ball (SGD), so that it can break out of these local minima. However, even with momentum, our algorithm might still get caught up in local minima if the learning rate (step size) is too small, whereas with too large a step size the algorithm might step entirely past all minima. This can be avoided by using an adaptive learning rate, such that the learning rate is set to be as large as possible while minimising the volatility of the changes in loss. Performance during training was evaluated using mean squared error^72^. MLPs were trained on the feature vector containing the SSVEP amplitudes at each EEG channel and relevant frequency (Fig. 3g).

#### K-Nearest neighbours (KNN)

Another classification approach which does not rely on linear boundaries between classes is K-nearest neighbour classification (KNN). This simple but powerful approach classifies each new sample by using a distance metric to find the class most common in the sample’s *k-*nearest neighbours in feature-space amongst the test set^52^. Using a 10-fold cross validation approach to hyperparameter optimization, we found that the optimal value for the number of neighbours (*k)* was 1. We applied KNN to the feature vector containing the SSVEP amplitudes at each EEG channel and relevant frequency using the *fitcknn* function from the MATLAB statistics and machine learning toolbox (Fig. 3d). Distance between data points was assessed using Euclidean distance.

### Behavioural results

Overall, participants were able to perform the task successfully. Predictably, the distractor present trials (accuracy *M* = 62.17%, *SD* = 16.46) proved more difficult than the distractor absent trials (accuracy *M* = 87.74%, *SD* = 14.07, *t*_29_ = 10.67, *p* < 0.001). This was also reflected in participants’ reaction times (RTs), which were faster for the distractor absent trials (RT *M* = 0.58 s, *SD* = 0.06) than for the distractor present trials (RT *M* = 0.64 s, *SD* = 0.08, *t*_29_ = −5.09, *p* < 0.001).

### SSVEP Amplitudes

**Fig. 4:**
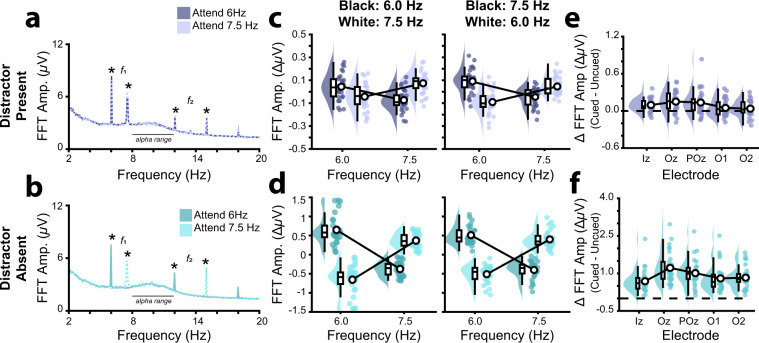
Feature-based attention effects on SSVEPs. (**a,b**) Grand average frequency spectra generated by submitting the average of all trials when participants were cued to attend dots flickering at 6.0 Hz and 7.5 Hz to an FFT, for (**a**) distractor present and (**b**) distractor absent trials. Black asterisks mark the SSVEPs at the first and second harmonics of the flicker frequencies. Black bars mark the frequencies at the alpha range. Note that while SSVEPs are evoked at the same frequency and phase across trials, endogenous alpha activity varies in phase across trials and is therefore not strongly visible in these trial average frequency spectra. (**c,d**) Raincloud plots of grand average SSVEPs at 6.0 Hz and 7.5 Hz when attention was cued toward each of these flicker frequencies, for (**c**) distractor present and (**d**) distractor absent trials^74^. SSVEPs are shown separately for the black: 6.0 Hz, white 7.5 Hz, and black: 7.5 Hz, white: 6.0 Hz conditions. Each participant’s marginal means (SSVEP amplitude at each flicker frequency) have been subtracted from their cell means before plotting, to clearly show the direction of individual effects. (Note that all reported statistics were performed on the untransformed data.) (**e, f**) Raincloud plots of the SSVEP attention effect (SSVEP_Cued Dots_ - SSVEP_Uncued Dots_) at each recorded electrode, for (**e**) distractor present and (**f**) distractor absent trials^74^. Raincloud plots show kernel density plots over the data (violin plots), individual data points for each participant (point cloud), and box plots indicating the quartiles of these data.

During distractor present trials, SSVEPs were evident at both 6.0 Hz and 7.5 Hz, as expected (Fig. 4a). To assess the effects of the attentional cue on SSVEP amplitude for the distractor present condition, we submitted SSVEP amplitude to a 2 × 2 × 2 repeated-measures ANOVA with SSVEP frequency (6.0 Hz, 7.5 Hz), flicker frequency of cued dots (6.0 Hz, 7.5 Hz) and colour-flicker frequency condition (black: 6.0 Hz and white: 7.5 Hz, black: 7.5 Hz and white: 6.0 Hz) as factors. There was no significant three-way interaction, such that the effect of cued frequency on SSVEP amplitudes at each frequency did not differ across colour-flicker frequency conditions (F(1, 232) = 0.03, p = 0.871, η_p_^2^ < 0.001, Fig. 4c). As expected, SSVEP amplitudes at each frequency differed according to the flicker frequency of the cued dots (F(1, 232) = 4.42, p = 0.037, η_p_^2^ < 0.019), such that SSVEP amplitudes at 6.0 Hz were significantly larger when 6.0 Hz was cued (M = 1.47, 95% C I[1.28, 1.65]) versus un-cued (M = 1.33, 95% CI [1.17, 1.50]; t_29_ = 11.88, p < 0.001), and SSVEP amplitudes at 7.5 Hz were larger when 7.5 Hz was cued (*M* = 1.05, 95% CI [0.93, 1.17]) versus un-cued (*M *= 0.93, 95% CI [0.81, 1.05]; t_29_ = 4.44, p < 0.001). This effect of cue on SSVEP amplitudes was relatively evenly distributed across the occipito-parietal electrode array (Fig. 4e)^6^.

During distractor absent trials, SSVEPs were only present at the cued frequency, as expected (Fig. 4b). To assess the effects of the attentional cue on SSVEP amplitude for the distractor absent condition, we submitted SSVEP amplitude to a 2 × 2 × 2 repeated-measures ANOVA with SSVEP frequency (6.0 Hz, 7.5 Hz), flicker frequency of presented dots (6.0 Hz, 7.5 Hz) and colour-flicker frequency condition (black: 6.0 Hz/ white: 7.5 Hz, black: 7.5 Hz/ white: 6.0 Hz) as factors. As for the distractor present trials, this analysis revealed that there was no significant three-way interaction, such that the effect of presented frequency on SSVEP amplitudes at each frequency did not differ across colour-flicker frequency conditions (F(1, 232) = 1.59, p = 0.208, η_p_^2^ = 0.007, Fig. 4d). As expected, SSVEP amplitudes at each frequency differed according to the flicker frequency of the presented dots (F(1, 232) = 404.04, p < 0.001, η_p_^2^ = 0.635), such that SSVEP amplitudes at 6.0 Hz were significantly larger when 6.0 Hz was presented (*M* = 1.30, 95% CI [1.08, 1.51]) versus not presented (*M* = 0.13, 95% CI [0.11, 0.15]; t_29_ = 11.49, p < 0.001), and SSVEP amplitudes at 7.5 Hz were significantly larger when 7.5 Hz was presented (*M* = 0.90, 95% CI [0.77, 1.03]) versus not presented (*M* = 0.13, 95% CI [0.11, 0.15]; t_29_ = 11.88, p < 0.001). This effect of presentation on SSVEP amplitudes was relatively evenly distributed across the occipito-parietal electrode array (Fig. 4f)^6^.

### Classification accuracy

Our dataset was collected to determine which pre-processing steps, extracted features and classification algorithms are best suited to discriminate between attended and unattended frequency tagged visual features. To this end, we sought to classify the single-trial EEG data using a number of different classification algorithms and training parameters to provide an initial benchmark for future work.

#### Flicker frequency

**Fig. 5:**
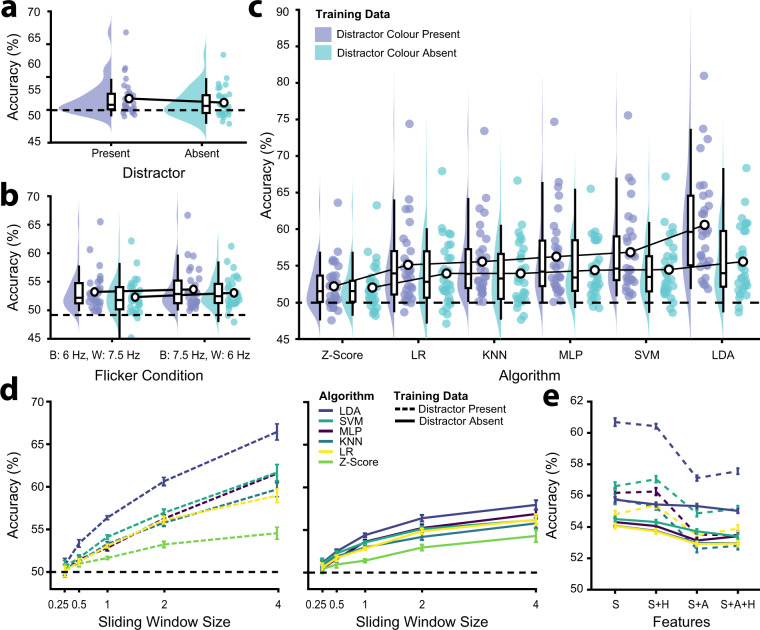
Classification accuracy for the attended feature (black dots, white dots) on the distractor present trials, calculated for each participant using trial-based 10-fold cross validation for different training parameters. (**a**) Raincloud plot of the average classification accuracy for classifiers trained using data from trials in which the distractor was present and absent^74^. (**b**) Raincloud plot of the average classification accuracy for classifiers trained using data from trials in each flicker frequency condition (black: 6.0 Hz and white: 7.5 Hz, black: 7.5 Hz and white: 6.0 Hz), for the distractor present and absent training data. Note that for the distractor absent trials, only the target frequency was presented. (**c**) Raincloud plot of the average classification accuracy for each classification algorithm, for the distractor present and absent *training* conditions. (**d**) Average classification accuracy for each classification algorithm trained on data from the distractor present and distractor absent conditions, for each of 5 sliding window sizes ranging from 0.25 s to 4.00 s. (**e**) Average classification accuracy for the LR, MLP, LDA, SVM, and KNN classification algorithms trained on each combination of training features (simple, simple + harmonic, simple + alpha, simple + alpha + harmonic), according to whether distractor present or absent training data were used.

The pairing of flicker frequency and colour was counterbalanced on a trial-by-trial basis, such that on half of the trials, black dots flickered at 6.0 Hz and white dots flickered at 7.5 Hz, and on the remaining trials the pairing was reversed. Given there are large individual differences in SSVEP amplitudes across frequencies^26^, classifiers were trained separately for these two conditions and classification accuracies were taken as the average result across the two flicker frequency conditions (Fig. 5b). Note that under either condition, participants were still equally likely to be cued to attend to either the black or white dots. Before assessing how the training parameters affected this average classification accuracy, we evaluated whether the specific pairing of flicker frequency and dot colour affected classification. Student’s *t*-tests revealed that there was no significant difference in classification accuracy across the two flicker conditions, both for classifiers trained on trials whether the distractor was present (black 6.0 Hz, white 7.5 Hz: *M* = 53.22, 95% CI [51.95, 54.49]; black 7.5 Hz, white 6.0 Hz: *M* = 53.66, 95% CI [52.31, 55.03]; *t*_29_ = −1.15, *p* = 0.261) and absent (black 6.0 Hz, white 7.5 Hz: *M* = 52.31, 95% CI [51.14, 53.49]; black 7.5 Hz, white 6.0 Hz: *M* = 53.03, 95% CI [51.96, 54.10]; *t*_29_ = −1.84, *p* = 0.076).

#### Presentation type

Flickering dots were presented either individually (distractor absent) or concurrently with distractors. Practical BCI and neurofeedback applications requiring real-time classification of feature-selective attention would be likely to involve at least two features/frequencies. However, we sought to determine whether training data free of noise from distractors might increase classification accuracy during selectivity. We therefore trained the classifiers on distractor present and absent data and tested these classifiers on the distractor present data (Fig. 5a). To assess how this and other training parameters affected the accuracy of classification of the attended feature, we submitted classification accuracy to a 2 × 5 × 6 repeated measures ANOVA with training data distractor condition (present, absent), sliding window size (0.25 s, 0.50 s, 1.0 s, 2.0 s, 4.0 s) and algorithm (Z-Score, LDA, LR, SVM, MLP, KNN) as factors. Overall, training on the distractor present condition (*M* = 54.07, SD = 3.61) resulted in significantly better classification accuracy than training on the distractor absent condition (*M* = 52.91, SD = 2.91, *F*(1, 1740) = 31.80, *p* < 0.001, η_p_^2^ = 0.018), suggesting that there was no benefit to training on distractor absent data. Further, the effects of sliding window size (*F*(4, 1740) = 16.59, *p* < 0.001, η_p_^2^ = 0.037) and algorithm (*F*(5, 1740) = 3.96, *p* = 0.001, η_p_^2^ = 0.011) also differed across the two different presentation types. As such, we evaluated the effects of these factors separately for the two different presentation types.

#### Classification algorithm

We applied a number of different classification algorithms (*Z*-Score, LDA, LR, SVM, MLP, KNN) to determine which colour of frequency-tagged dots participants were attending at any given moment (Fig. 5c). For classifiers trained on the distractor *present* condition, there was a significant main effect of the classification algorithm (*F*(5, 174) = 5.63, *p* < 0.001, η_p_^2^ = 0.139). We therefore evaluated the performance of each classifier in this condition using a Student’s *t*-test against the baseline z-score approach (Bonferroni corrected α = 0.01 [0.05/5]). In order of performance from best to worst, average classification accuracy across all sliding window sizes and training features was as follows: LDA (*M* = 60.58, 95% CI [57.93, 63.23]; *t*_29_ = 8.11, *p* < 0.001), SVM (*M* = 56.84, 95% CI [54.70, 58.98]; *t*_29_ = 4.92, *p* < 0.001), MLP (*M* = 56.23, 95% CI 54.08, 58.39]; *t*_29_ = 4.19, *p* < 0.001), KNN (*M* = 55.55, 95% CI 53.63, 57.46]; *t*_29_ = 3.65, *p* = 0.001), LR (*M* = 55.11, 95% CI [53.06, 57.16]; *t*_29_ = 3.07, *p* = 0.005), and z-score (*M* = 53.15, 95% CI [51.39, 54.91]).

As described above, training the classification algorithms on data from the condition where the distractor was *absent* from the display resulted in significantly worse performance than training on the distractor present condition. Unlike for the distractor present condition, there was no significant main effect of algorithm for classifiers trained on distractor absent data (*F*(5, 174) = 1.25, *p* = 0.286, η_p_^2^ = 0.035; Fig. 5c). A Student’s *t*-test against chance (50%) demonstrated that on average (across all other conditions), training on distractor absent data did result in classification accuracies greater than chance (*M* = 54.15, 95% CI [52.56, 55.74]; *t*_29_ = 5.35, *p* < 0.001). However, given the low classification accuracy for this condition compared with distractor present, we would not recommend training on distractor absent data for the classification of feature-selective attention. The maximum classification accuracy (across sliding window sizes and frequency combinations) achieved for each participant and each training condition is displayed in Table 6.

**Table 6:** Maximum classification accuracy achieved for each participant for each classifier and presentation type (across sliding window sizes and frequency combinations).

SubID	Train: Distractor Present						Train: Distractor Absent					
	Z-Score	LDA	LR	SVM	MLP	KNN	Z-Score	LDA	LR	SVM	MLP	KNN
sub-01	51.55	61.62	57.59	61.24	56.23	56.16	53.38	55.72	55.22	58.20	56.12	56.54
sub-02	60.28	62.07	59.00	63.87	59.72	59.88	58.57	63.45	61.69	63.52	61.74	61.75
sub-03	62.75	82.63	71.93	77.36	78.67	75.41	62.26	65.35	62.95	60.45	65.10	61.97
sub-04	64.48	83.84	71.07	75.14	78.61	72.26	63.74	67.37	65.52	62.01	64.25	61.40
sub-05	54.06	57.65	52.06	54.32	54.42	56.06	54.05	52.70	56.75	56.88	54.32	57.54
sub-07	55.88	66.74	60.97	61.65	61.20	57.42	55.42	59.31	59.00	58.62	57.14	57.94
sub-08	41.83	57.75	54.13	52.75	51.96	54.51	44.45	50.25	46.23	47.44	50.91	47.73
sub-09	51.49	52.34	47.98	53.33	53.12	53.49	52.54	53.25	52.17	54.07	52.49	52.55
sub-10	61.24	70.86	62.21	65.33	67.41	64.47	62.34	63.04	62.89	61.27	63.85	63.06
sub-11	75.71	90.89	82.30	85.60	86.98	83.06	76.63	73.50	75.43	72.65	70.53	71.40
sub-12	50.58	68.91	55.26	63.41	62.22	57.28	53.20	55.88	52.59	51.03	54.00	50.18
sub-13	53.20	55.35	55.36	50.44	53.57	54.32	54.28	55.38	56.61	56.04	56.12	57.18
sub-14	53.59	69.07	59.39	61.64	65.52	59.92	50.54	55.06	51.63	55.81	56.48	52.66
sub-15	46.94	59.13	58.28	58.08	58.57	56.74	45.35	49.98	48.11	51.04	50.21	48.02
sub-16	48.92	56.72	50.52	56.27	55.67	52.75	47.66	59.25	52.15	53.56	56.70	52.21
sub-17	46.00	81.56	70.78	74.08	75.54	69.94	46.34	52.07	50.64	51.61	50.51	51.46
sub-18	56.91	58.19	54.62	59.03	56.40	57.06	53.83	54.57	52.51	54.26	55.49	54.38
sub-20	56.77	65.98	54.39	55.45	55.45	53.17	53.08	57.33	55.45	56.35	56.47	54.48
sub-21	51.53	60.85	55.93	56.06	55.25	50.01	52.76	52.45	53.56	51.72	51.54	51.96
sub-22	52.31	73.84	62.05	65.35	63.98	61.11	54.12	53.93	54.18	52.62	53.62	50.43
sub-23	51.58	72.26	61.61	69.68	69.20	66.97	51.70	65.39	59.63	60.90	60.89	58.03
sub-24	48.35	59.16	52.57	54.38	56.12	49.35	46.91	56.09	50.79	52.44	54.95	51.31
sub-25	52.27	62.27	56.14	56.33	59.75	58.47	52.68	56.28	54.09	53.61	55.86	54.89
sub-26	65.55	76.26	69.44	70.07	72.81	70.46	62.56	65.10	66.65	66.65	66.16	67.57
sub-27	49.78	66.05	57.78	59.53	61.73	60.02	49.38	52.96	54.90	56.39	56.36	56.75
sub-28	53.59	75.41	59.63	64.25	64.47	61.24	51.56	66.83	53.65	53.58	55.95	53.95
sub-29	62.37	69.21	65.14	68.56	63.71	64.71	63.14	64.21	63.59	62.67	59.81	63.15
sub-30	48.88	56.73	52.69	56.52	56.86	57.02	49.17	52.26	52.18	51.03	53.08	49.68
sub-31	56.01	64.27	57.83	54.87	55.53	53.80	56.55	58.65	57.78	58.18	58.01	59.21
sub-32	52.60	66.37	53.32	61.04	60.24	58.45	50.85	61.35	54.32	56.26	55.23	53.75
Mean	54.57	66.80	59.40	62.19	62.36	60.18	54.30	58.30	56.43	56.70	57.13	56.10

#### Sliding window size

Overall, there was a significant main effect of sliding window size for classification algorithms trained on both the distractor present (*F*(4, 145) = 20.76, *p* < 0.001, η_p_^2^ = 0.364) and distractor absent trials (*F*(4, 145) = 9.49, *p* < 0.001, η_p_^2^ = 0.207), such that larger sliding window sizes resulted in higher accuracy (Fig. 5d). Notably, this effect was larger for distractor present training. The greater accuracy with increasing sliding window size is unsurprising, as larger sliding windows by nature include more data, and the longer time-periods likely smooth out fluctuations in the strength of attentional selectivity and data quality. Interestingly, though, the increase is not linear, and can be well characterised by an inverse exponential function. Using MATLAB’s *fittype* and *fit* functions, which apply the method of least squares, we fitted an inverse exponential function to the classification accuracy curve:

1 $$ACC=a\left(1-{e}^{-s{\rm{(}}T-i{\rm{)}}}\right)$$

Where *ACC* is classification accuracy, *T* is the number of training trials, *a* is the asymptote, *s* is the scaling factor and *i* is the *x*-axis intercept^73^. This model suggests that for distractor present trials, the average classification accuracy (across all algorithms) has an asymptote at 64.67% (95% CI [62.27, 67.72), and classification at 99% of this asymptote should be achievable with a 11.25 s sliding window size. By contrast, for distractor absent trials, the average classification accuracy (across all algorithms) has an asymptote at 55.87% (95% CI [53.76, 58.00), and classification at 99% of this asymptote was reached at the 3.5 s sliding window size. Thus, there are diminishing returns on increasing the sliding window size, which is an important consideration when designing BCI or neurofeedback protocols. The protocols must weigh accuracy against sensitivity and control; while a 20 second sliding window is likely to yield a relatively accurate representation of the current target of attentional selectivity, this does not allow for a readout of short term fluctuations in attention and is likely to be too slow for practical BCI control.

#### Training frequency range

The classification algorithms (with the exception of the baseline z-score approach) were trained on four different combinations of frequencies extracted from the Fast Fourier Transformed EEG signal (Fig. 5e). These included; ‘simple’: spectral power at the two flicker frequencies, ‘simple + harmonic’: spectral power at the first and second harmonics of the flicker frequencies, ‘simple + alpha’: spectral power at the flicker frequencies and from endogenous alpha oscillations, ‘simple + alpha + harmonic’: spectral power at all evaluated frequencies. Overall, the contributions of these different frequencies did not appear to make a meaningful difference to classification accuracy (Fig. 5e). A 2 × 5 repeated measures ANOVA with training data distractor condition (present, absent), and training features (simple, simple + harmonic, simple + alpha, simple + alpha + harmonic) as factors suggested that the effect of training features did not interact with the effect of distractor condition (*F*(3, 232) = 0.58, *p* = 0.626, η_p_^2^ = 0.007). Indeed, there was no significant main effect of training features (*F*(3, 232) = 7.78, *p* < 0.078, η_p_^2^ = 0.029. Nevertheless, an individual differences approach revealed that the LDA classifier trained on distractor present data, which performed highest overall, performed best for 15 participants (out of 30) using the simple flicker frequencies, for 14 participants using these frequencies and their second harmonics, for 0 participants using these frequencies and the alpha frequencies, and for 1 participant using a combination of the flicker frequencies, their second harmonics, and the alpha frequencies. Using the classification accuracy for this classifier on the 4 s sliding window epochs, there was a significant difference between classification accuracy using the lowest performing frequencies for each participant (*M* = 60.25%, *SD* = 8.14, Range = 49.12–85.16%) as opposed to using the highest performing frequencies for each participant (*M* = 66.80%, *SD* = 9.52, Range = 52.34–90.89%; *t*_29_ = 10.45, p < 0.001). This suggests there might be some benefit to tailoring the classification features for each individual participant.

## Usage Notes

The experimental task, which is written in MATLAB, requires the installation of Psychtoolbox-3 (http://psychtoolbox.org/) to run. The EEG data are stored in the Brain Vision format. For MATLAB, we recommend using the Fieldtrip Toolbox (https://www.fieldtriptoolbox.org/) to load these data. See “*FeatAttnClassification\AnalysisScripts\Main_Decoding.mlx*” and the “*FeatAttnClassification\AnalysisScripts\Functions\get_eeg.m”* function for specific examples of how to load these data into MATLAB. To load the EEG data into Python, we recommend the MNE-python package (https://mne.tools/stable/index.html)/). Behavioural data for each participant are stored in.mat files. This native MATLAB format can be read into MATLAB using the *load* function. For Python, the behavioural files are saved in HDF5 format and can be read using the h5py package (https://pypi.org/project/h5py/).

The analysis scripts were written in MATLAB using The Statistics and Machine Learning Toolbox. Then functions required to run these scripts are included in *“AnalysisScripts\Functions”*. For an overview of how EEG data were processed and how the machine learning classification was performed, please see the MATLAB Live notebook *“FeatAttnClassification\AnalysisScripts\Main_Decoding.mlx”*. This is available in the MATLAB format “.mlx”, as well as “.pdf” and “.html” formats.

## Data Availability

The full repository containing all data and code folders described above is available through the Open Science Framework (https://osf.io/c689u/)^45^. The analysis code and experimental task code are also available on Github (https://github.com/air2310/FeatAttnClassification).
